# Exploratory benchtop study evaluating the use of surgical design and simulation in fibula free flap mandibular reconstruction

**DOI:** 10.1186/1916-0216-42-42

**Published:** 2013-06-24

**Authors:** Heather Logan, Johan Wolfaardt, Pierre Boulanger, Bill Hodgetts, Hadi Seikaly

**Affiliations:** 1 Institute for Reconstructive Sciences in Medicine, 1W-02, 16940-87 Avenue Misericordia Community Hospital, Edmonton, AB T5R 4H5, Canada; 2 Department of Computing Science, Athabasca Hall, Room 411 Edmonton, University of Alberta, Edmonton, AB T6G 2E8, Canada; 3 Speech Pathology and Audiology 2–16, Corbett Hall Edmonton, University of Alberta, 116 St. and 85 Ave, Edmonton, AB T6G 2G4, Canada; 4 Department of Otolaryngology-Head & Neck Surgery, 8440 - 112th Street, 1E4.34 WMC, Edmonton, AB T6G 2B7, Canada

**Keywords:** Virtual surgical planning, Surgical design and simulation, Fibula free flap mandibular reconstruction, Digital registration

## Abstract

**Background:**

Surgical design and simulation (SDS) is a useful tool to help surgeons visualize the anatomy of the patient and perform operative maneuvers on the computer before implementation in the operating room. While these technologies have many advantages, further evidence of their potential to improve outcomes is required. The present benchtop study was intended to identify if there is a difference in surgical outcome between free-hand surgery completed without virtual surgical planning (VSP) software and preoperatively planned surgery completed with the use of VSP software.

**Methods:**

Five surgeons participated in the study. In Session A, participants were asked to do a free-hand reconstruction of a 3d printed mandible with a defect using a 3d printed fibula. Four weeks later, in Session B, the participants were asked to do the same reconstruction, but in this case using a preoperatively digitally designed surgical plan. Digital registration computer software, hard tissue measures and duration of the task were used to compare the outcome of the benchtop reconstructions.

**Results:**

The study revealed that: (1) superimposed images produced in a computer aided design (CAD) software were effective in comparing pre and post-surgical outcomes, (2) there was a difference, based on hard tissue measures, in surgical outcome between the two scenarios and (3) there was no difference in the time it took to complete the sessions.

**Conclusion:**

The study revealed that the participants were more consistent in the preoperatively digitally planned surgery than they were in the free hand surgery.

## Background

Fibular reconstruction of the mandible is a challenging surgical procedure undertaken to correct tumour resection, traumatic injury or congenital deformities of the lower jaw. In the past, a significant discontinuity defect of the jaws presented the surgeon with a difficult, if not insurmountable reconstruction challenge. With the advent of microvascular reconstruction, reconstruction of the mandible became a more predictable procedure. As a consequence, the fibula free flap became the workhorse for mandibular discontinuity defect reconstruction and has become the preferred method for mandibular reconstruction because of the adequate bone stock length, and acceptance of dental implants [1-4]. With a predictable mandibular reconstruction technique available, new options for oral rehabilitation arose with osseointegrated implants but this came with the need for improved precision and accuracy of the reconstruction.

Reconstruction of the mandible can potentially result in negative sequelae in oral functions such as deglutition, speech, mandibular movements, mastication and control of saliva [5]. These sequelae can be mitigated by preoperative planning of the operative procedure through surgical design and simulation. These processes have the potential of reducing the major negative changes in the patient’s quality of life that can lead to low self-confidence and negative self perception [6-8].

In recent years, surgical design and simulation (SDS) has become a widely available and reliable tool for preoperative surgical planning. Digital imaging technology is a useful tool to help surgeons visualize the defect of the patient and perform operative maneuvers on the computer before implementation in the operating room [9]. While these technologies have many advantages, there continues to be resistance and limitations to their clinical use which include financial, technical and practical challenges.

Many studies have discussed the benefits of VSP technologies and medical models in maxillofacial reconstruction [9-14] but very few have confirmed whether there is a difference between free hand surgery and preoperatively virtually planned surgery, and if so, what the difference is between surgical strategies.

The purpose of the present study was to examine whether there was a difference, in the context of surgical simulation, between free hand surgery completed without VSP and preoperatively planned surgery completed with the use of VSP.

## Methods

Five surgeons with experience in microvascular reconstruction were asked to participate in the study by means of purposive sampling due to the limited number of head and neck surgeons in Edmonton, Alberta, as well as the limitations of the scope of the project.

**Figure 1:**
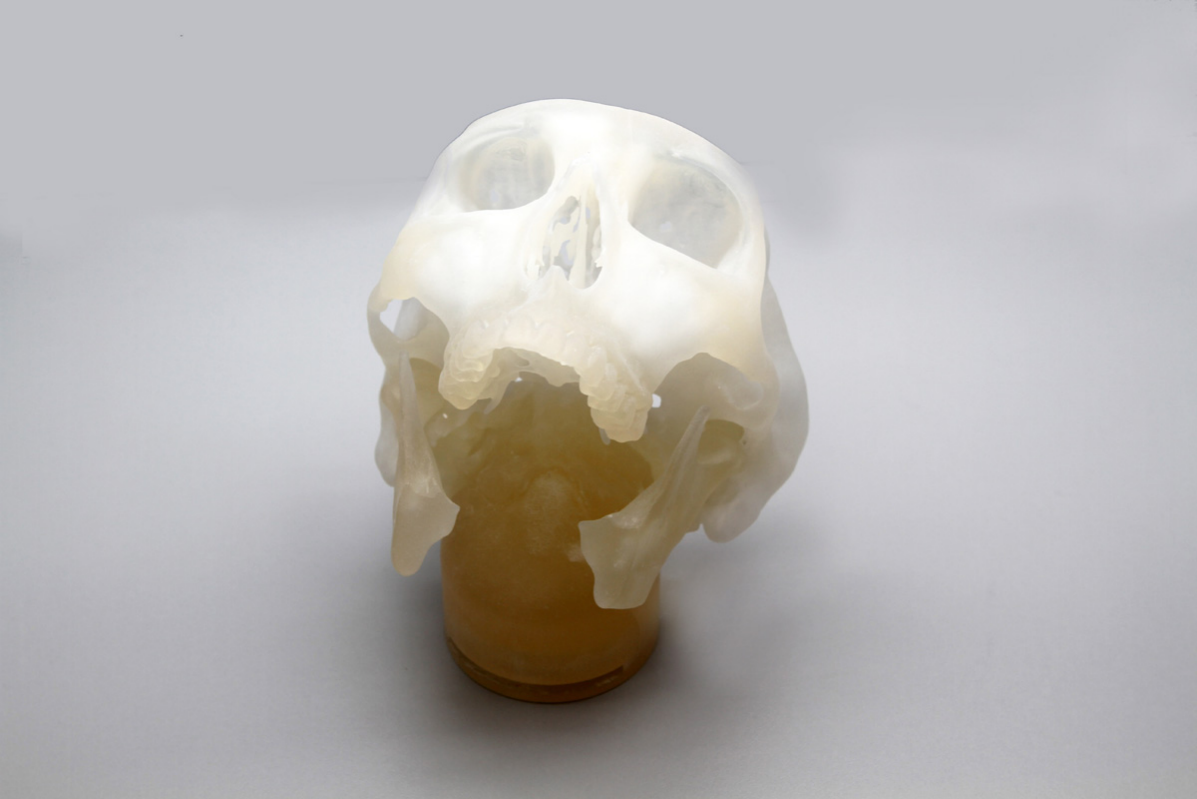
Design of the benchtop surgery scenario with the remaining of the resected mandible in place.

The present study was a repeated measures study composed of two benchtop sessions (A and B). In Session A, participants were asked to do a free-hand reconstruction of a standardized 3d printed mandible with an angle-to-angle defect using a standardized 3d printed fibula (Figure 1). No less than four weeks later, in Session B, the participants were asked to reconstruct the same standardized rapid prototyped mandible, but in this case using a preoperatively digitally designed surgical plan including a patient-specific external fixator and a patient-specific bone cutting guide developed by the researcher.

**Figure 2:**
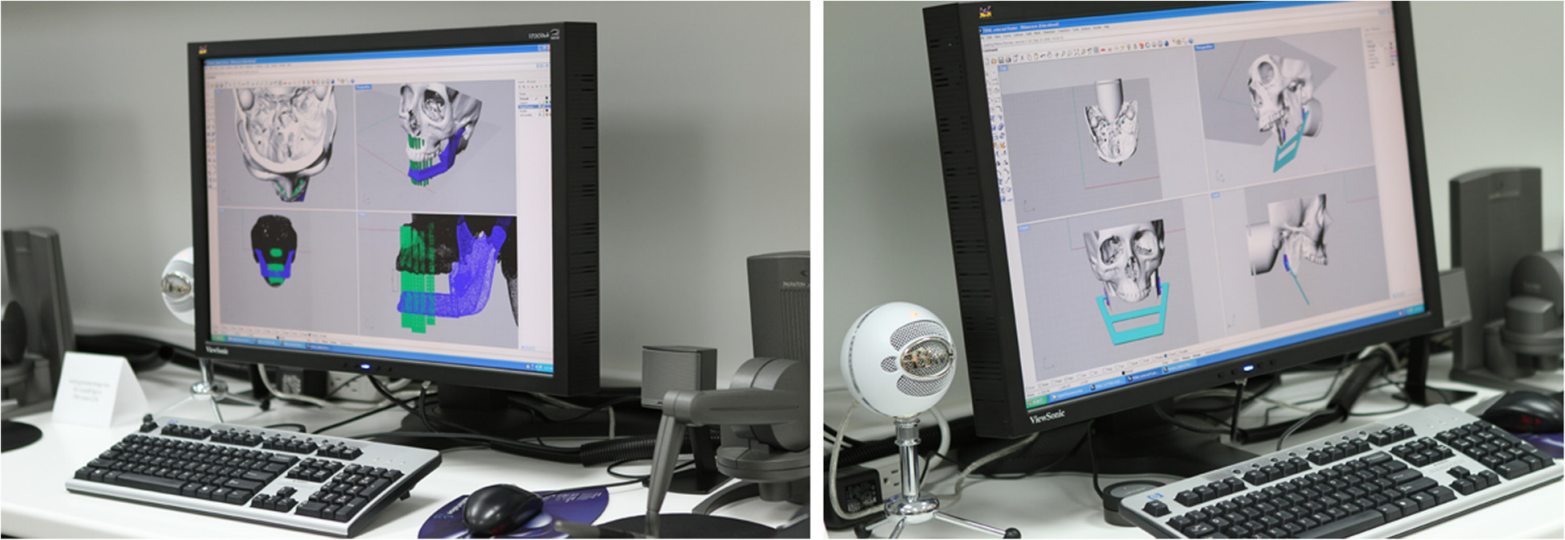
**On-screen digital plan. **Representation of the on-screen digital plan of the mandible reconstruction reviewed with the participants at the beginning of Session B.

**Figure 3:**
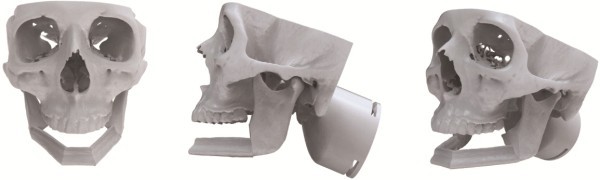
**Digital mandibular reconstruction. **Mandibular reconstruction with maxilla. From left to right; front view, side view and perspective view.

**Figure 4:**
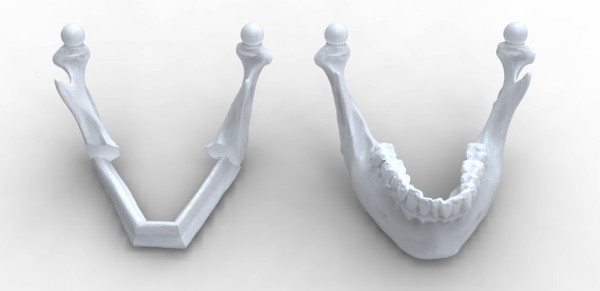
**Control mandibles. **The left image is the optimal digital reconstruction of the mandible and the right is the native mandible.

**Figure 5:**
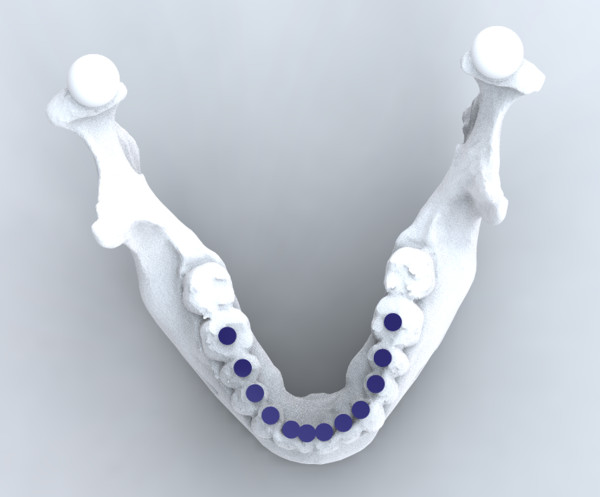
**Optimal implant locations. **Digital representation of the potential optimal implant locations in relation to the native mandible.

**Figure 6:**
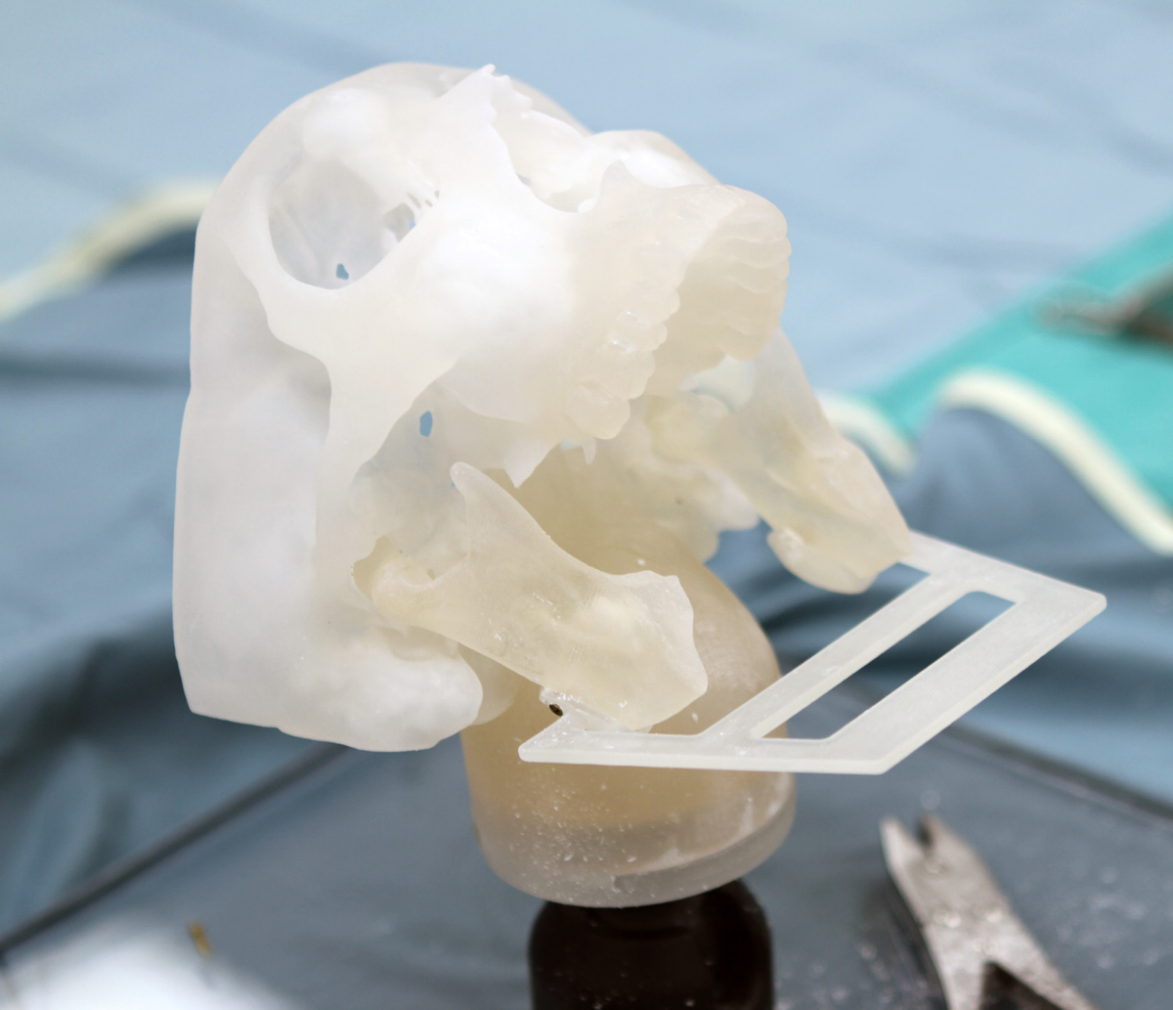
**External fixator. **Representation of the patient specific external fixator screwed onto the rami.

**Figure 7:**
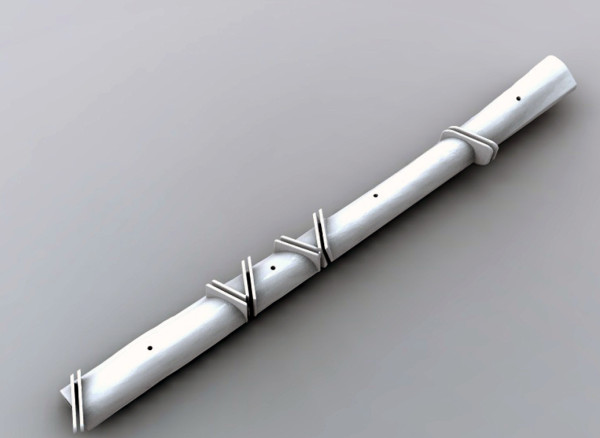
**Fibula cutting guide. **Top view of the patient specific fibula cutting guide.

Each participant completed Sessions A and B individually in the Medial Modeling Research Laboratory (MMRL) at the Institute of Reconstructive Sciences in Medicine (iRSM) at the Misericordia Community Hospital in Edmonton, Alberta, Canada. At the beginning of Sessions A and B, all participants were given the same list of instructions explaining the guidelines and objectives to follow (see Additional files 1 and 2). The researcher reviewed the steps of the procedure, all 3d printed models and all the surgical and mechanical tools with the participants to ensure that they had a clear understanding of the task. In Session B all participants reviewed an on screen digital plan of the reconstruction with the researcher (Figure 2). During the review, the researcher discussed the optimal mandibular reconstruction (Figure 3 and Figure 4), the potential optimal implant placement in relation to the native mandible (Figure 5), the external fixator design (Figure 6) and the cutting guide design (Figure 7) with the participants.

Sessions A and B were compared in two sets of virtual comparisons using automatic digital registration and a series of hard tissue manual measurements.

It was decided that the study should focus on medical models instead of human patients due to ethical considerations and limitations in assigning patients to different and potentially limiting treatment plans. It was also an important feature of this study to maintain a consistent surgical process and anatomy among all participating surgeons. A one-month transition period between Session A and B was set in place to avoid potential motor memory learning and training.

### Benchtop design

The digital reconstruction design of the mandible was developed in collaboration with a head and neck surgeon and a maxillofacial prosthodontist. The researcher met with the two clinicians in order to design the reconstruction with optimal implant location, optimal placement of the fibula segments in relation to the native mandible and optimal height in relation to the occlusal plane.

### Outcome measures

**Figure 8:**
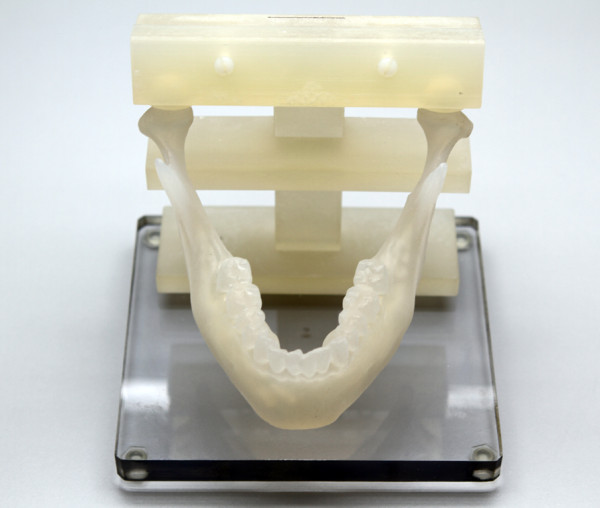
**Scanning jig. **Jig design for the CT scanning with the mandible in place.

**Figure 9:**
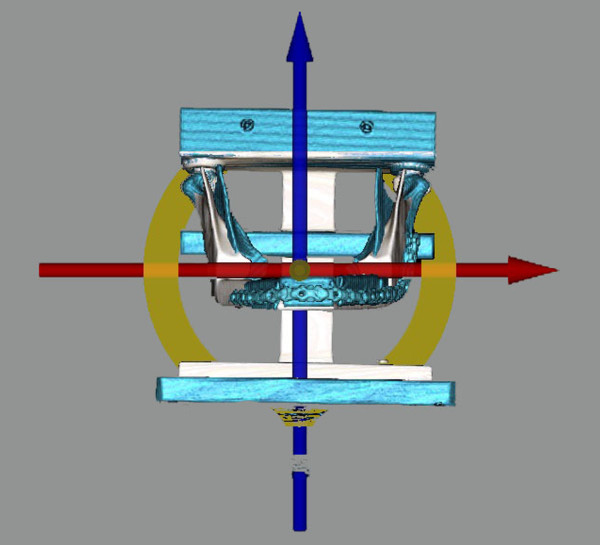
**The adjust tool. **The adjust tool was used in order to precisely match the two models if the registration point procedure was not exact.

Following Sessions A and B, all models that were reconstructed were scanned using Computer Tomography (CT) scanning technology. A jig was designed to hold the mandible reconstructions in place during CT scanning and to ensure that all scans were done in a precise and consistent orientation (Figure 8). The data from the CT scan were transferred into the software program InVivoDental 5.0 Anatomy Imaging Software (Anatomage Inc. San Jose, CA. USA) where a custom thresholding procedure was performed generating a polygonal model. The converted data were then transferred into the software programs Rhinoceros 4.0 (McNeel North America, Seattle WA, USA), Rapidform 2006 (INUS Technology, Inc. Seoul, Korea) and InVivoDental 5.0. The optimal mandible reconstruction was aligned to the reconstructions of Session A and B as well as aligning the reconstructions of Session A to the reconstructions of Session B in order to assess whether the software programs were an effective tool for evaluating surgical outcome. In both programs (InVivoDental 5.0 and Rapidform), a minimum of three registration points common to both models of interest were identified. The software then automatically aligned the two models of interest. In Rapidform 2006, a global automated registration was completed in order to match the models more precisely, whereas in InVivoDental 5.0, the adjust tool (Figure 9) was used in order to precisely match the two models if the registration point procedure was not exact.

Thirteen hard tissue measures were used in the present study (Additional file 3: Table S1). All measurements were recorded by the researcher on five separate days in randomized order in order to verify the reliability and repeatability of the measuring technique and to reduce intra-rater bias.

## Results and discussion

Additional file 4: Table S2 represents the mandible reconstructions completed by all participants.

### Software superimposed images

One of the objectives of the present study was to evaluate whether digital registration software is an effective tool for evaluating surgical outcome. Both InVivoDental 5.0 and Rapidform 2006 software programs were able to produce very similar results. It was considered that InVivoDental 5.0 was more user friendly compared to Rapidform 2006 and was more efficient in producing the images due to the adjust tool (Figure 9) which allowed the user to move the superimposed objects in any direction and any rotation in order to register the objects precisely. Rapidform 2006 often took two or more attempts to acceptably register the two images and did not have an adjustment tool. Additional file 5: Table S3 represents a series of superimposed images produced by the InVivoDental 5.0 software. The first column represents the mandibles reconstructed in Session A (blue) superimposed onto the digital reconstruction control model (white). The second column represents the mandibles reconstructed in Session B (blue) superimposed onto the digital reconstruction control model (white). The third column represents the mandibles reconstructed in Session A superimposed onto the mandibles reconstructed in Session B (colors vary, see table for description).

### Hard tissue measures

#### ***Manual measures reliability analysis***

A reliability analysis was performed to assess whether the measures taken on five separate days was reliable. All measures had a strong mean correlation between each measure verifying the reliability and repeatability of the technique.

#### ***Hard tissue manual measure analysis***

The second objective was to assess whether there was a difference in surgical outcome between Session A and Session B based on the hard tissue manual measures. Each measure was compared to the control mandible or the digital reconstruction based on the optimal anticipated outcome. All manual measure averages were subtracted from the corresponding control mandible averages which gave the deviation from the control for each measure of each participant. These values were then used in the analysis.

The exploratory data analysis (EDA) technique was used to analyze all measure comparisons due to the small sample size of the study [15].

Of the thirteen manual measures, four displayed large differences between Session A and B. The four measures were the left gonial angle, the right gonial angle, the inter-coronoid process length and the fibula crest length. Additional file 6: Table S4 outlines the descriptive statistics of the four measures.

#### ***Reconstruction time (in minutes)***

Each participant was timed during Session A and Session B in order to assess whether one technique took less time than the other technique. The timing began as soon as the surgeon picked up a tool and the timing ended as soon as they verbally said to the researcher that they were finished. The mean time to complete Session A was 79.75 minutes while the mean time to complete Session B was 74.25 minutes.

## Discussion

### Software superimposed images

The superimposition of Session A with the control mandible, Session B with the control mandible and Session A with Session B was undertaken to view all the possible comparisons. Both InVivoDental 5.0 and Rapidform 2006 software programs generated informative images which allowed the viewer to quickly understand where the differences were when comparing the mandibles. The images showed that overall Session B appeared to have more accurate reconstructions that were closer to the control compared to Session A. Session B also appeared to be more consistent in the reconstructions compared to Session A which showed more variability among participants.

The disadvantage of these software programs was that they were unable to produce any information other than images. The present study was limited in its anatomy used for analyses. A future study using patient cases may reveal more applications of the InVivoDental 5.0 software program.

## Model study manual measures

### Measures showing a potential difference between Session A and Session B

#### ***Measure 1: left gonial angle and Measure 2: right gonial angle***

One of the objectives for the participating surgeons was to design the reconstruction for 15 mm dimension between the upper surface of the fibula and the occlusal plane to accommodate implant abutment and superstructure components. This objective was determined by the gonial angle. For the left gonial angle measure, Session A had a range of 19.81 mm while Session B had a range of 4.87 mm. This is a difference of 14.94 mm. The stem and leaf graph and the boxplot for both the right and left gonial angle showed that the participants were more accurate and consistent in Session B than they were in Session A.

In Session A, participants had to gauge the 15 mm distance by eye and use a ruler to determine the spatial positioning of the fibula segments. In Session B, the participants had the same task to accomplish but had the advantage of a patient-specific cutting guide which provided the participants with the proper angle and length of the cuts of the fibula. They also had the digital reconstruction reference model as a visual reference to compare their cuts to and to bend the reconstruction plate around. Both of these tools contributed to the accuracy of the left and right gonial angle and the consistency of the participants in Session B.

Clinically the distance between the upper surface of the fibula and the occlusal plane can have a large impact on the functional outcome for the patient. One of the disadvantages of the fibula is the limited height of the bone (rarely more than 15 mm) which is about half of the native mandible [16]. This presents a problem for prosthetic rehabilitation and wearing ossesointegrated-implant retained dentures [4]. The recommended ratio for crown:fixture length is 1:1.5 [17,18]. When the distance between the upper surface of the fibula and the occlusal plane is too large, patients often need long abutments (7 to 10 mm in some cases creating a crown:fixture ratio of 1:1.21) or a superstructure of excessive vertical dimension to compensate for the large vertical discrepancy. This has potential to endanger implant stability [18]. Hence, it is important in preoperative planning to consider the height and angle of the fibula in relation to the intended occlusal plane before reconstruction. On the other hand, excessive thickness of the bone can lead to a poor degree of mouth opening, which can affect eating, speech, efficiency of lip closure and oral functions [5].

The left and right gonial angles conveyed an important functional outcome measure for fibula free flap mandibular reconstruction and showed a large difference between the free-hand technique and the surgical design technique. This anatomical measure is an essential measure to consider for future analysis of patient outcome and possible future studies evaluating surgical outcome.

#### ***Measure 4a: inter-coronoid process width***

Session A had a much larger median compared to Session B as well as a larger range. This showed that the surgeons were more consistent in Session B as well as closer to the control mandible compared to Session A. The patient specific external fixator used in Session B assisted the surgeons in maintaining the inter-coronoid process width and is most likely the contributing factor to the overall better performance in Session B. The proper inter-coronoid process length is an important anatomical outcome which can contribute to many problems in the future for the patient if it is either too long or too short.

#### ***Measure 6: fibula crest length***

The boxplot of Measure 6 revealed a slight overlap but visually overall and by looking at the descriptive statistics, there appears to be a difference between the outcome of Session A and Session B. Session A had a range of 19.94 mm while Session B had a range of 7.85 mm for the fibula crest length measurement. This is a difference of 12.09 mm. The patient specific cutting guide, the external fixator and the 3d printed model of the digital reconstruction of the mandible used in Session B all contributed to the superior results of the fibula crest lengths in Session B. Overall the measure of the fibula crest length received the best results from the surgical design and the digital reconstruction.

Clinically, the fibula crest length may have a significant impact on the geometric and functional outcome of the patient. The fibula crest length is important for osseointegrated implants and dental rehabilitation for the patient. The length and geometry of the fibula crest has a direct impact on the patient’s jaw relationship, which may affect mastication and speech.

#### ***Reconstruction time (in minutes)***

The recording of the time it takes to complete the benchtop model sessions revealed that there is no difference in the time it takes the participants to complete one session compared to the other. Using the external fixator, the cutting guide and the digital reconstruction of the mandible reference model did not produce any changes in the length of time to complete the reconstructions.

## Conclusion

The objective of the model study was to assess whether there is a difference in surgical outcome between free-hand surgery completed without VSP as opposed to preoperatively planned surgery completed with the use of VSP. The digital registration tools used to produce visual images of the differences between Session A and B revealed that CAD software tools can be a helpful and an effective tool in understanding and comparing pre and post surgical outcomes. The hard tissue manual measurements revealed that the participants were more consistent, based on optimal surgical outcome, in the preoperatively digitally planned surgery than they were in the free hand surgery. The exploratory data analysis technique revealed that the left and right gonial angle, the inter-coronoid process length and the fibula crest length all had the greatest difference between the two surgical techniques. The measures also revealed that the use of the patient-specific guide and external fixator was helpful in obtaining results that were more accurate in relation to the control mandible. The comparison of the time (in minutes) it took the participants to complete each session revealed that there is no difference in time to complete one session compared to the other. The present study also revealed positive feedback based on the experiences of the participants after Session B of the benchtop study. From the three outcome measures, it appeared that SDS produces positive and effective differences in surgical outcome compared to free-hand surgery completed without SDS.

## Abbreviations

(SDS): Surgical design and simulation; (VSP): Virtual surgical planning; (CAD): Computer aided design; (MMRL): Medial modeling research laboratory; (iRSM): Institute of reconstructive sciences in medicine; (EDA): Exploratory data analysis; (CT): Computer tomography.

## Competing interests

The authors declare that they have no competing interest.

## Authors’ contributions

HL, JW, HS, BH and PB all conceived the study, participated in its design and coordination and drafted the manuscript. HS participated in the conception and design of the surgical components of the study. BH participated in the statistical design and statistical analysis of the study. PB participated in the conception of the digital measuring techniques and appropriate software. The present study was one of three studies done to complete a thesis for a Master of Science in Rehabilitation Science focusing in Surgical Design and Simulation (SDS). JW, HS, BH and PB all participated as supervisory committee members for the MSc of HL. All authors read and approved the final manuscript.

## Authors’ information

Heather Logan (HL) - MSc, BDes

HL is a Surgical Design Simulationist at the Institute for Reconstructive Sciences in Medicine (iRSM). She completed her Master of Science in Rehabilitation Science with a focus in Surgical Design and Simulation in 2011 at the University of Alberta. HL divides her time between clinical work in facial prosthetics, surgical design and simulation and research. Her research involves developing and refining surgical design methods and developing digital processes for facial prosthetic fabrication.

Dr. Johan Wolfaardt - BDS, MDent (Prosthodontics), PhD.

JW is a Director of the Institute of Reconstructive Sciences in Medicine (iRSM) and is appointed as a Full Professor in the Faculty of Medicine and Dentistry, University of Alberta, Canada. His clinical and research interests are in the area of maxillofacial prosthetics with particular emphasis in the area of head and neck reconstruction, osseointegration and treatment outcomes. JW has led the development of the research program at COMPRU. His research interests involve treatment outcomes, digital technologies in head and neck reconstruction and biomechanics of osseointegrated implants. JW has published over 80 papers in refereed journals and contributed to a variety of texts. He has lectured both nationally and internationally on maxillofacial prosthetics, osseointegration in head and neck reconstruction, challenges of introduction of advanced digital technology, knowledge work, teamwork and quality management. JW is elected to the Boards of the International Society of Maxillofacial Rehabilitation, the American Academy of Maxillofacial Prosthetics and the International College of Prosthodontists.

Dr. Hadi Seikaly - MD, FRCSC.

HS is a Professor of Surgery in the Department of Surgery (University of Alberta), Director of the Division of Otolaryngology Head and Neck Surgery, and the Zone Section Head for Otolaryngology Head and Neck Surgery. In addition, he is presently the president of the University Hospital Medical Staff Society, serves on several local, national, and international administrative committees and is on the Executive Council of the Canadian Society of Otolaryngology. HS graduated from the University of Toronto medical school and completed his residency training at the University of Alberta in Otolaryngology Head and Neck Surgery. He then obtained fellowship training at the University of Texas Medical Branch in advanced head & neck oncology, microvascular reconstruction and facial cosmetic surgery. Dr. Seikaly returned to the University of Alberta as an attending in the division of Otolaryngology, department of surgery in 1996 where he has been active in teaching, patient care, and research.

HS continues to have a large practice dedicated to head, neck and skull base oncology and reconstruction. His research interests include functional surgical and reconstructive outcomes, microvascular head and neck reconstruction, submandibular gland transfer and medical modeling as it applies to the head and neck region.

He has published more than 50 papers in peer reviewed journals and numerous chapters.

Dr. Pierre Boulanger – Ph.D., P.Eng.

PB worked for 18 years at the National Research Council of Canada as a senior research officer where his primary research interests were in 3D computer vision, rapid product development, and virtualized reality systems. He now holds a double appointment as a professor at the University of Alberta in the Department of Computing Science and in the Department of Radiology and Diagnostic Imaging (Faculty of Medicine). PB is the Director of the Advanced Man Machine Interface Laboratory as well as the scientific director of the Alberta Radiological Visualization Center. His main research topics and teachings are on virtualized reality systems and medical imaging. He is also a Principal Investigator for Stereo IPTV at TRLabs. PB has published more than 260 scientific papers in various journals and conferences. PB is on the editorial board of two major academic journals as well as on many international committees and frequently gives lectures on rapid product development and virtualized reality. On the commercial side, PB is the President of PROTEUS Consulting Inc., an Alberta-based consulting firm specialized in Virtual Reality Applications.

Dr. Bill Hodgetts – Ph.D.

BH obtained his B.A. in Psychology and his M.Sc. in Audiology at the University of Western Ontario. He received his Ph.D. in Rehabilitation Sciences at the University of Alberta. BH is an Associate Professor in the Department of Speech Pathology and Audiology at the University of Alberta where he teaches in the areas of hearing science/audiology and research methods and statistics. He has a joint appointment with the Institute for Reconstructive Sciences in Medicine (iRSM), where he is program director of Bone Conduction Amplification. BH’s research involves developing and refining the selection, verification, and validation of fitting procedures for BAHA (bone anchored hearing aid). He also has a research interest in bone anchored hearing aids and noise exposure from MP3 Players.

## Supplementary Material

Additional file 1 Utility of Digital Surgical Simulation Planning and Solid Free Form Modeling in Fibula Free Flap Mandibular Reconstruction: Benchtop study: session A. Click here for file

Additional file 2 Utility of Digital Surgical Simulation Planning and Solid Free Form Modeling in Fibula Free Flap Mandibular Reconstruction: Benchtop study: session B. Click here for file

Additional file 3: Table S1 Thirteen hard tissue measures. Click here for file

Additional file 4: Table S2 Session A and Session B results. Click here for file

Additional file 5: Table S3 Series of superimposed images produced in InVivoDental 5.0 software. Click here for file

Additional file 6: Table S4 Descriptive statistics of measures 1-4. Click here for file
