# Supplementary material for: Exploratory benchtop study evaluating the use of surgical design and simulation in fibula free flap mandibular reconstruction
Source: J Otolaryngol Head Neck Surg. 2013 Jun 24;42(1):42. doi: 10.1186/1916-0216-42-42 (PMC3729729; doi:10.1186/1916-0216-42-42)
Supplement: Additional file 2 — Utility of Digital Surgical Simulation Planning and Solid Free Form Modeling in Fibula Free Flap Mandibular Reconstruction: Benchtop study: session B. [file 1916-0216-42-42-S2.pdf]

Utility of Digital Surgical Simulation Planning and Solid Free Form Modeling in Fibula Free Flap Mandibular Reconstruction

Principal Investigator:
Dr. Johan Wolfaardt BDS, MDent, PhD

Researcher:
Heather Logan, BDes

Collaborator:
Dr. Hadi Seikaly, M.D. FRCS

BENCHTOP STUDY: SESSION B

MODELS:

Maxilla

Left fibula: 9cm removed from each end

Surgical design reference model of virtually planned reconstruction

Patient specific external fixator

Angle to Angle defect to be reconstructed

Reference fibula

Patient specific fibula cutting guide

MATERIALS:

1 x 2.0mm 4 x 20 x 4 double angled Locking Plate

12 x 2.0mm titanium Locking screw Plus drive 10mm long

20 x 2.0mm titanium cortex screw Plus drive 10mm long

1 x set of instruments to implant above implants

1 x saw for preparation of acrylic bones

1 x saw blade

1 x cordless drill

1 x drill bit

1 x ruler

OBJECTIVES:

1. Reconstruct to original form of the native mandible
2. Design the reconstruction to optimize aesthetic and functional outcome
3. Design the reconstruction for oral rehabilitation with osseointegrated implants
4. Design the reconstruction for a 15mm dimension between the upper surface of the fibula and the occlusal plane to accommodate implant abutment and superstructure components.
5. Review the on screen digital plan of the reconstruction with research coordinator.

5. Screw external fixator to remaining native mandible for proper ramus orientation.

6. Use surgical design model of the virtually planned reconstruction to bend the double angled Locking Plate.

7. Screw patient specific cutting guide to fibula and follow guide for proper angles and dimensions of fibula osteotomies.

INSTRUCTIONS:

1. Your time will be recorded but please take as much time as necessary to achieve the objectives.
2. You may change the orientation of the model.
3. Follow surgical design as close as possible to ensure best possible outcome.
