# Supplementary material for: Exploratory benchtop study evaluating the use of surgical design and simulation in fibula free flap mandibular reconstruction
Source: J Otolaryngol Head Neck Surg. 2013 Jun 24;42(1):42. doi: 10.1186/1916-0216-42-42 (PMC3729729; doi:10.1186/1916-0216-42-42)
Supplement: Additional file 3: Table S1 — Thirteen hard tissue measures. [file 1916-0216-42-42-S3.doc]

**Table 1** **Thirteen hard tissue measures**

| **Measure #** | **Image of measure** | **Measure #** | **Image of measure** |
| --- | --- | --- | --- |
| **1** Left gonial angle | 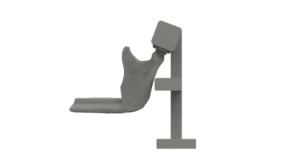 | **5b** Ramus to ramus length outside of the jig. | 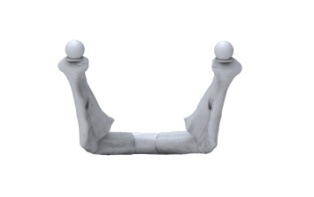 |
| **2** Right gonial angle | 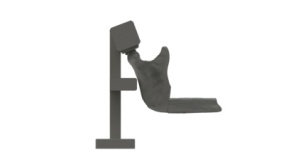 | **6** Fibula crest length | 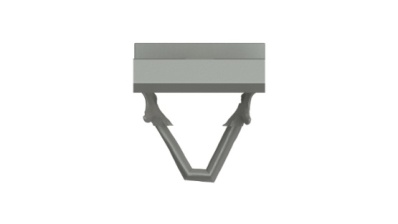 |
| **3** Pogonion | 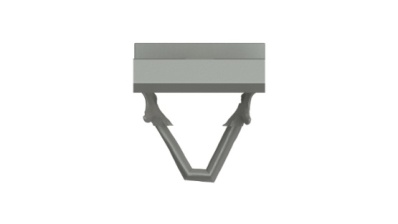 | **7** Right ramus angle from front view | 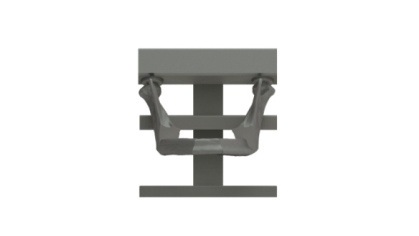 |
| **4a** Inter-coronoid process width | 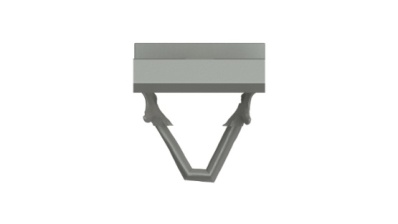 | **8** Left ramus angle from front view | 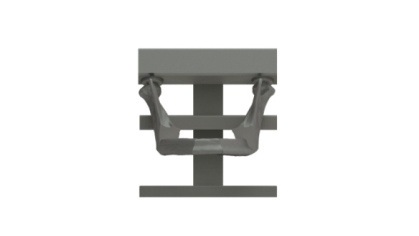 |
| **4b** Inter-coronoid process width outside of the jig. | 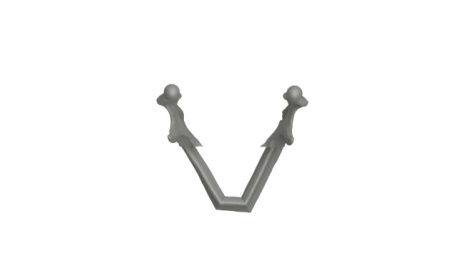 | **9** Number of fibula segments used to complete the reconstruction | 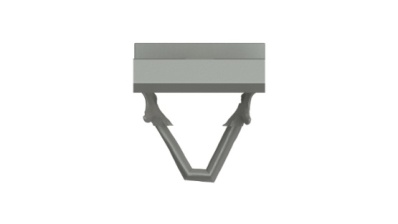  2  3  1 |
| **4c** Ball joint to ball joint distance outside of the jig. | 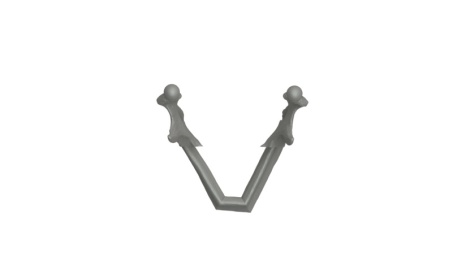 | **10** Intersegment distance of fibula | 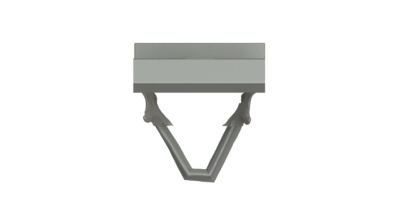 |
| **5a** Ramus to ramus length inside of the jig. | 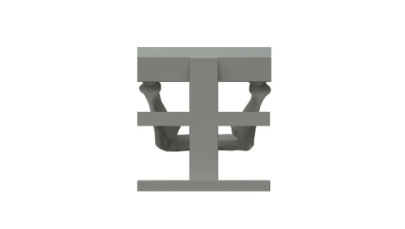 |  |  |
