# Supplementary figures and images for: Exploratory benchtop study evaluating the use of surgical design and simulation in fibula free flap mandibular reconstruction
Source: J Otolaryngol Head Neck Surg. 2013 Jun 24;42(1):42. doi: 10.1186/1916-0216-42-42 (PMC3729729; doi:10.1186/1916-0216-42-42)

**Table 2** **Session A and Session B results**

| **Participant** | **Session A (left) and Session B (right)** |
| --- | --- |
| **1** | **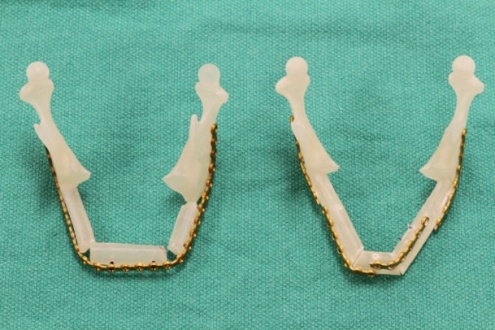** |
| **2** | **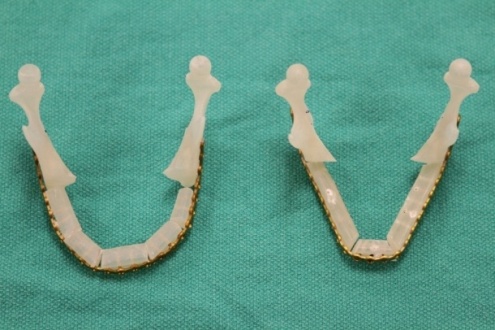** |
| **3** | **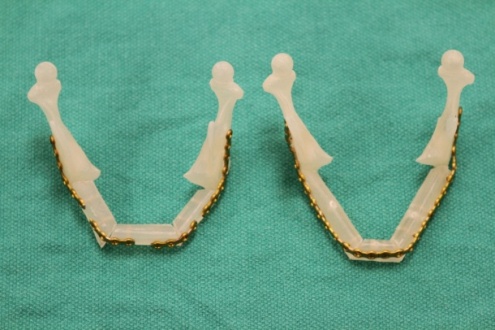** |
| **4** | **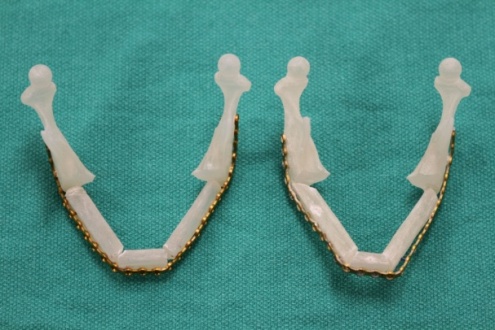** |
| **5** | **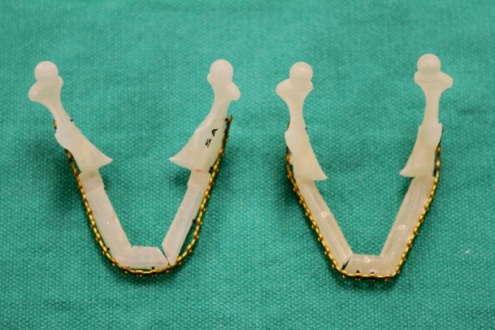** |

Supplement: Additional file 4: Table S2 — Session A and Session B results. [file 1916-0216-42-42-S4.doc]
