# Supplementary material for: Exploratory benchtop study evaluating the use of surgical design and simulation in fibula free flap mandibular reconstruction
Source: J Otolaryngol Head Neck Surg. 2013 Jun 24;42(1):42. doi: 10.1186/1916-0216-42-42 (PMC3729729; doi:10.1186/1916-0216-42-42)
Supplement: Additional file 5: Table S3 — Series of superimposed images produced in InVivoDental 5.0 software. [file 1916-0216-42-42-S5.doc]

**Table 3** **Series of superimposed images produced in InVivoDental 5.0 software**

| **Session A with the Control Mandible** | **Session B with the Control Mandible** | **Session A and Session B Superimposed** |
| --- | --- | --- |
| 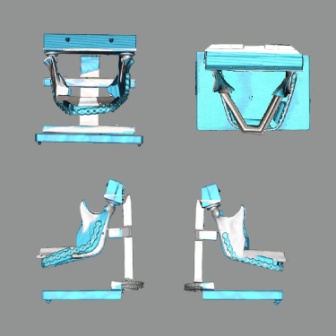  Participant #1 White = control, Blue = recon | 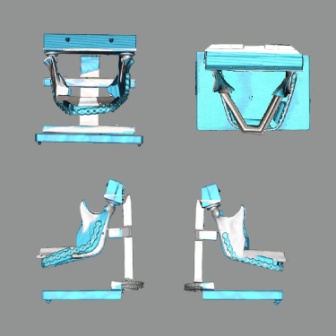  Participant #1 White = control, Blue = recon | 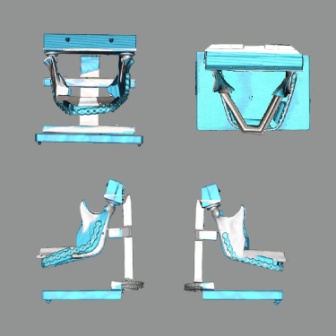  Participant #1 White = Session A, Blue=Session B |
| 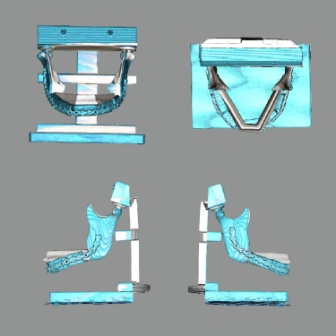  Participant #2 White = control, Blue = recon | 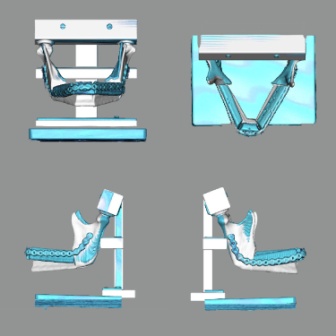  Participant #2 White = control, Blue = recon | 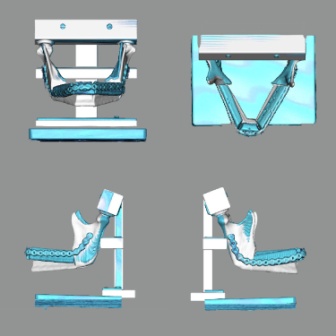  Participant #2 White = Session A, Blue= Session B |
| 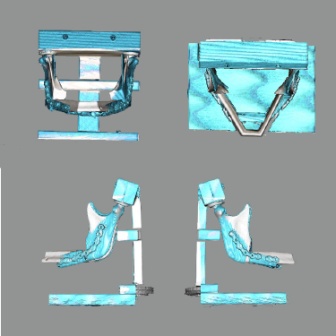  Participant #3 White = control, Blue = recon | 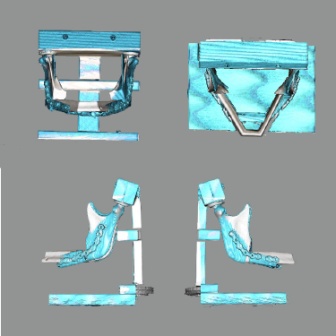  Participant #3 White = control, Blue = recon | 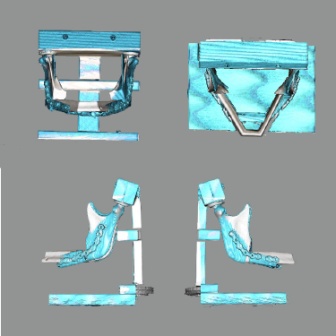  Participant #3 White = Session A, Blue= Session B |
| 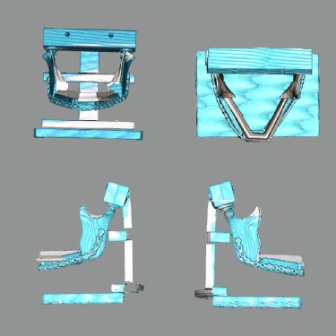  Participant #4 White = control, Blue = recon | 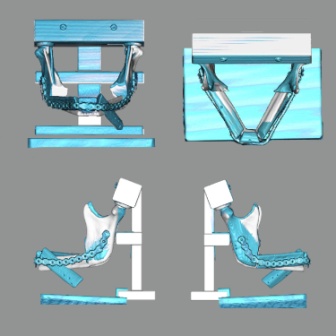  Participant #4 White = control, Blue = recon Note: one fibula segment screw fell out during scanning process | 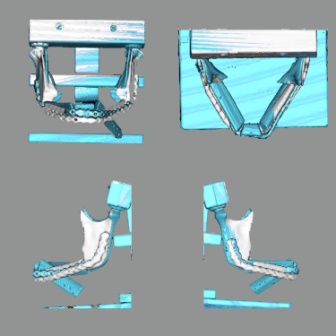  Participant #4 White = Session A, Blue = Session B Note: one fibula segment screw fell out during scanning process |
| 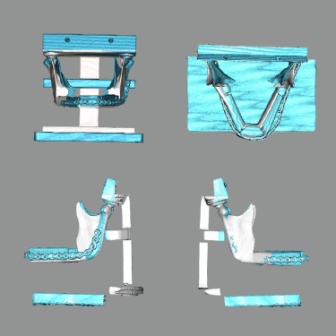  Participant #5 White = control, Blue = recon | 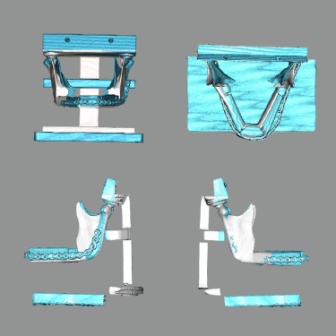  Participant #5 White = control, Blue = recon | 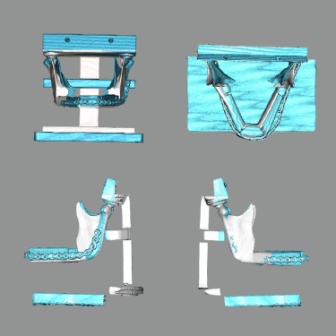  Participant #5 White = Session A, Blue= Session B |
