# Supplementary material for: Exploratory benchtop study evaluating the use of surgical design and simulation in fibula free flap mandibular reconstruction
Source: J Otolaryngol Head Neck Surg. 2013 Jun 24;42(1):42. doi: 10.1186/1916-0216-42-42 (PMC3729729; doi:10.1186/1916-0216-42-42)
Supplement: Additional file 6: Table S4 — Descriptive statistics of measures 1-4. [file 1916-0216-42-42-S6.doc]

Table S4. Descriptive statistics of measures 1-4

| **Measure** | **Descriptive Statistics**  (median and range of the average deviation from the digital reconstruction control model) | **Visual Display of the results** |
| --- | --- | --- |
| Measure 1:  Left Gonial Angle | **Session A**  Median: 12.94mm  Range: 19.81mm  **Session B:**  Median: 3.75mm  Range: 4.87 | **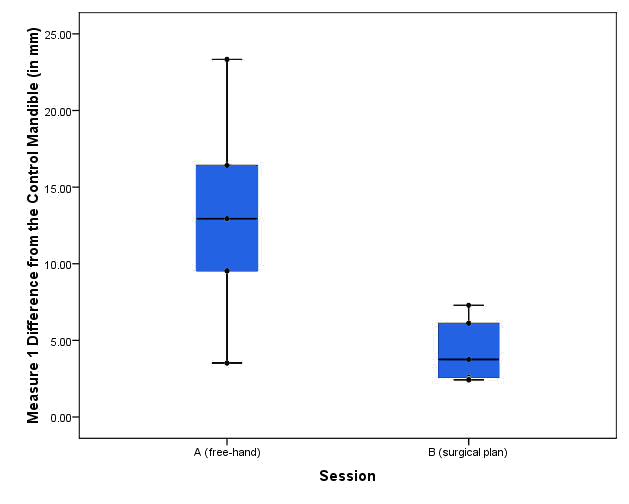** |
| Measure 2:  Right Gonial Angle | **Session A**  Median: 8.78mm  Range: 9.71mm  **Session B:**  Median: 5.50mm  Range: 8.00mm | **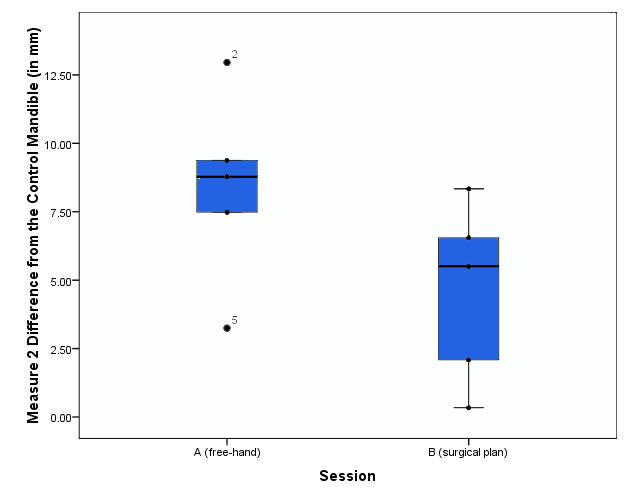** |
| Measure 4a:  Inter-coronoid process width | **Session A**  Median: 10.84mm  Range: 20.12mm  **Session B:**  Median: 2.55mm  Range: 6.48mm | **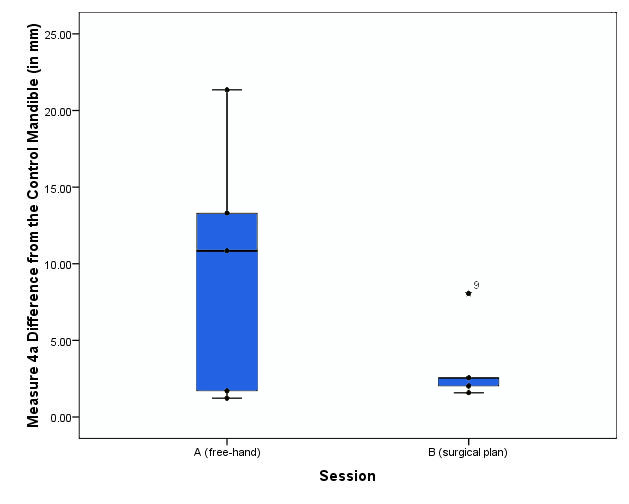** |
| Measure 6:  Fibula crest length | **Session A**  Median: 8.12mm  Range: 20.45mm  **Session B:**  Median: 4.85mm  Range: 7.06mm | **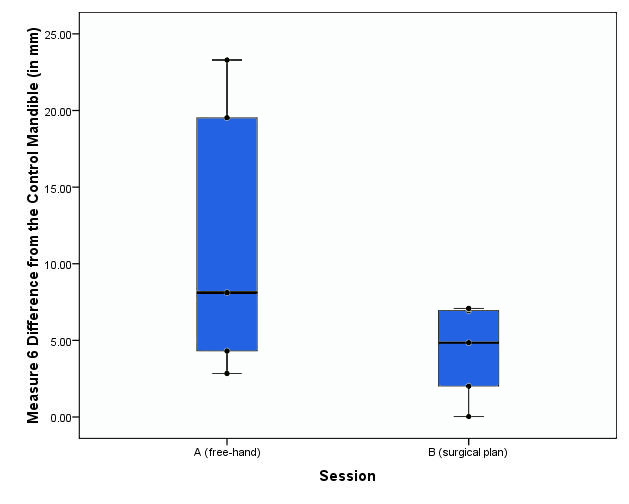** |
